# Dual function of heterotrophic ammonia-oxidizing bacteria in facilitating maize compensatory growth under limited rewatering after drought

**DOI:** 10.1186/s12896-025-01006-z

**Published:** 2025-07-11

**Authors:** Qiang Lv, Ruo-Yu Hao, Xiao-Ling Wang, Li-Ju Zhou, Lin Qi, Peng Song

**Affiliations:** 1 College of Agronomy, Henan University of Science and Technology, Luoyang, Henan 471003 China; 2 School of Traditional Chinese Pharmacy, China Pharmaceutical University, Nanjing, Jiangsu 210000 China

**Keywords:** Dual function, Heterotrophic ammonia-oxidizing bacteria, Maize, Cytokinin, Rhizosphere soil nitrification, Post-drought limited rewatering, Compensatory growth

## Abstract

Water scarcity threatens global food security, making drought resilience in crops like maize crucial. In response to this challenge, this study investigates the potential of heterotrophic ammonia-oxidizing bacteria (HAOB) to enhance maize compensatory growth under post-drought limited rewatering conditions. Specifically, we focus on the dual mechanism of HAOB in modulating cytokinin synthesis and transport, aiming to develop an innovative agricultural biotechnology to support sustainable crop production. The *S2_8_1* HAOB strain was used across two experiments. Experiment 1 investigated varying NO_3_^−^ levels’ effects on cytokinin translocation from roots to leaves under limited rewatering. Experiment 2 combined NO_3_^−^ supplementation with HAOB inoculation to assess HAOB’s twofold function in promoting compensatory growth under limited rewatering. The results showed that optimal NO_3_^−^ levels (20–30 mmol·L^− 1^ for limited rewatering) enhanced maize growth, root-to-shoot cytokinin translocation, and leaf cytokinin levels under limited rewatering. Notably, inoculation with HAOB outperformed these effects, demonstrating a more robust impact on cytokinin delivery and plant growth. This confirmed HAOB’s twofold mechanism: Nitrification pathway – HAOB enhances rhizospheric NO₃⁻ availability, thereby stimulating cytokinin biosynthesis in roots and its translocation to leaves. Non-nitrification pathway – HAOB further promotes cytokinin translocation through mechanisms independent of soil NO₃⁻ increase. Sufficient rewatering increased rhizosphere nitrification rates, boosting root cytokinin translocation to leaves, thereby supported compensatory growth. Limited rewatering reduced rhizosphere nitrification, cytokinin translocation, and compensatory growth. However, HAOB overcame these constraints through its twofold function, enhancing cytokinin translocation and improving water use efficiency by more than fourfold, successfully promoting compensatory growth even under limited rewatering. Additionally, NO_3_^−^ supplementation alleviated some limitations by increasing rhizosphere NO_3_^−^, but HAOB inoculation proved more effective, highlighting its superior role. This twofold function of HAOB strain significantly elevated cytokinin levels in leaves, supporting compensatory growth under limited rewatering. This biotechnology offers high agricultural potential, particularly in water-scarce regions, by improving drought resilience and yield stability.

## Introduction

Globally, particularly in dryland regions, farmlands often experience short- or long-term water shortages, followed by irrigation or rainfall, leading to a phenomenon known as “post-drought rewatering”. During this process, crops previously inhibited by drought stress can exhibit accelerated growth upon rewatering, sometimes achieving or even surpassing the growth levels of non-stressed crops [1, 2]. This compensatory growth enhances crop water use efficiency without significantly impacting overall growth and yield, making it an effective approach for both water conservation and yield improvement. Research has demonstrated that this theory has been successfully applied in regulated deficit irrigation, deficit irrigation, supplementary irrigation, and rainwater-efficient utilization techniques in dryland agriculture, yielding significant economic benefits [3–5]. Limited rewatering, often associated with most moderate rainfall, deficit irrigation, is more common than sufficient rewatering, which typically results from regulated deficit irrigation or heavy rainfall. As a result, limited rewatering is more widespread and water-efficient in agricultural practices, underscoring the importance of research in this area to advance water conservation strategies in agriculture.

Water scarcity has emerged as a critical threat to global food production and security, heightening the urgency to enhance crop water use efficiency [6]. This study focuses on compensatory growth in crops following drought under limited rewatering conditions, aiming to advance water-efficient agricultural practices. Sufficient rewatering increases the crop photosynthetic rate, promoting post-drought compensatory growth [7], research highlights the pivotal role of root-sourced cytokinin in this process [2, 8]. Specifically, maize experiencing drought followed by sufficient rewatering shows an increase in cytokinin synthesis in the roots due to soil nitrate (NO_3_^−^). This cytokinin is then transported to the leaves, enhancing photosynthesis and promoting compensatory growth. Further research indicates that sufficient rewatering can stimulate soil nitrification [9, 10], increasing NO_3_^−^ content in the rhizosphere soil, which promotes root cytokinin synthesis and transport, thereby enhancing compensatory growth. The application of organic fertilizers also benefits soil nitrification and supports post-drought rewatering compensatory growth in plants [11]. Limited rewatering impairs photosynthesis, hindering compensatory growth [12]. However, there are few reports on how soil nitrification induces compensatory growth through enhanced cytokinin synthesis in roots under limited rewatering conditions.

Soil nitrification is primarily driven by microorganisms such as ammonia-oxidizing bacteria and archaea. Wang [13, 14] have shown that heterotrophic ammonia-oxidizing bacteria (HAOB) strains can colonize the maize rhizosphere. Sufficient rewatering of post-drought activates these HAOB, promoting nitrification and releasing more NO_3_^−^. This stimulates the upward transport of root-sourced cytokinin to the aerial parts, thereby promoting over-compensatory growth. However, under limited rewatering conditions, the interactions between water supply, HAOB activity, rhizosphere nitrification, and compensatory growth remain largely unexplored. Soil microorganisms are increasingly crucial in improving crop drought resistance and water use efficiency, making them a key focus of research to address water scarcity and boost crop yields [15–17]. Investigating the role of HAOB in regulating crop compensatory growth under limited rewatering is essential for advancing this research.

We propose that the mechanism by which HAOB promotes compensatory crop growth is not limited to enhancing soil NO₃⁻ release and stimulating root-derived cytokinin transport (the “nitrification pathway”). Under limited rewatering, nitrogen fertilizers alone are insufficient to achieve compensatory growth, highlighting the limitations of this approach [18]. We believe that HAOB offers an alternative pathway to overcome these constraints. Therefore, we hypothesize that combining both pathways can mitigate water supply limitations on cytokinin transport, thereby enabling compensatory growth and addressing current theoretical gaps.

To test this hypothesis, we used the HAOB strain *S2_8_1* and selected maize as the experimental crop. As the world’s third most cultivated crop, maize is highly responsive to drought and rewatering, making it well-suited for this study. We supplied an excess of NO_3_^−^ to the roots to assess the HAOB strain’s influence on cytokinin transport through the nitrification pathway. By combining NO_3_^−^ application and HAOB inoculation, we aimed to determine if HAOB stimulates cytokinin transport to leaves through dual action to achieve compensatory growth under limited rewatering.

## Materials and methods

### Experimental design

#### HAOB strain

We selected the *S2_8_1* heterotrophic ammonia-oxidizing bacterium (HAOB) from the genus *Serratia*, family *Rhizobiaceae*, preserved at the China Center for Type Culture Collection in Wuhan (CCTCC M2021374) and assigned the GenBank accession number ON667919. This strain is capable of growing in both *LB medium* and inorganic ammonia-oxidizing medium, but it exhibits faster and more robust growth in *LB medium*, indicating a preference for heterotrophic metabolism. Therefore, it is classified as a heterotrophic ammonia-oxidizing bacterium, as previously described by Wang et al. (2022). The inorganic liquid medium used for AOB isolation and cultivation consisted of 0.5 g (NH₄)₂SO₄, 0.75 g KH₂PO₄, 0.25 g NaH₂PO₄, 0.01 g MnSO₄·4 H₂O, 0.03 g MgSO₄·7 H₂O, and 5.0 g CaCO₃ dissolved in 1 L of distilled water, with the pH adjusted to 7.2. In this study, we inoculated the *S2_8_1* strain into this inorganic medium for subsequent experiments. This medium was chosen due to its low risk of contamination and its suitability for maintaining the strain’s ammonia-oxidizing activity. Its simplicity and effectiveness also facilitate experimental consistency and operability. When the culture was inoculated with *S2_8_1*, its number in the culture was about 12,300,000 cfu/m L.

To qualitatively verify the presence and activity of the *S2_8_1* ammonia-oxidizing bacterium (AOB) strain, the liquid culture medium was tested using Griess reagent, which detects nitrite ions—a byproduct of ammonia oxidation. Specifically, 0.5 mL of the liquid medium was placed onto a white porcelain colorimetric plate, followed by the addition of 0.1 mL of Griess reagent. The development of red, pink, or dark red coloration indicated the presence of nitrite, confirming the oxidation of ammonia and thereby the activity of the AOB strain. In this case, a distinct dark red color was observed, confirming effective ammonia oxidation by the *S2_8_1* strain.

This strain demonstrates minimal secretion of common plant hormones such as indole-3-acetic acid (IAA) and gibberellic acid (GA₃), but exhibits relatively strong cytokinin production. Zeatin riboside (ZR), a principal component of cytokinin, was quantified using commercial ZR assay kits (Shanghai Enzyme-linked Biotechnology Co., Ltd., Shanghai, China). Cultures of *S2_8_1* grown in inorganic medium for more than two weeks exhibited ZR concentrations as high as 28 ng/mL, while IAA and GA₃ levels remained below one-fourth of this value. Our previous study [13] similarly demonstrated that *S2_8_1* inoculation significantly enhanced ZR content in maize seedlings, with no notable impact on IAA or GA₃ levels.

#### Treatment setup for experiment 1 and 2

For our pot experiment, we selected the drought-tolerant maize cultivar ‘*Zhengdan 985*’ (*Zea mays* L.), conducted at the experimental farm of Henan University of Science and Technology in Luoyang, Henan Province, China (34°32´N, 112°16´E, 138 m above sea level). This area experiences an average yearly rainfall of 600 mm and a mean temperature of 14.1 °C.

On June 15, 2023, fifteen maize seeds were planted in each of the 400 plastic pots, each with a diameter of 20 cm, a height of 25 cm, and a capacity of 13.5 L of soil. Each pot contained 5.75 kg of brown soil with an organic carbon content of 23.6 g·kg⁻¹ and a total nitrogen level of 2.2 g·kg⁻¹. Maize seedlings emerged approximately six days after sowing. Another six days later, they were thinned, leaving five healthy plants per pot.

**Fig. 1:**
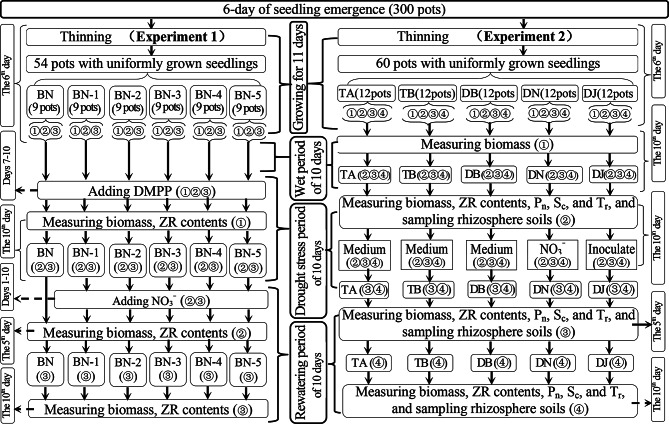
Schematic diagram of the experimental design. Experiment 1: BN to BN-5 represent limited rewatering treatments with 0 (water only), 10, 20, 30, 40, and 50 mmol·L⁻¹ NO₃⁻, respectively. Experiment 2: TA: well-watered control; TB: sufficient rewatering after drought; DB: limited rewatering; DN: well-watered + HAOB inoculation; DJ: limited rewatering + HAOB inoculation. Subgroups “①”–“④” indicate sequential sampling sets. DMPP (3,4-dimethylpyrazole phosphate) was used to inhibit soil nitrification. P_n_, S_c_, and T_r_ denote net photosynthetic rate, stomatal conductance, and transpiration rate. “Inoculate” and “Medium” refer to HAOB-inoculated and uninoculated culture solutions, respectively

On the eleventh day post-emergence, 54 and 60 pots with uniformly healthy seedlings were selected for Experiments 1 and 2, respectively. The subsequent 30-day period was divided into three 10-day phases: a wet phase (days 11–20), a drought phase (days 21–31), and a rewatering phase (days 32–42). Figure 1 presents the experimental timeline, treatment design, and measured indicators for both experiments.

#### Experiment 1

In Experiment 1, a 3,4-dimethylpyrazole phosphate (DMPP) solution (1.5 g·L^− 1^) was applied daily (30 mL per pot) to these 54 pots, beginning three days before the end of the drought period. Based on methods from earlier studies [19, 20], DMPP was employed to inhibit soil nitrification, limit NO_3_^−^ availability, and enhance the influence of exogenously added NO_3_^−^. These experiments aimed to evaluate the impact of varying NO_3_^−^ concentrations on maize growth and the synthesis and transport of cytokinins.

Fifty-four pots were assigned to six treatment groups (BN, BN-1, BN-2, BN-3, BN-4, and BN-5), each comprising nine pots. Each group was further divided into three subgroups of three pots, with each pot serving as a biological replicate. At the end of the drought period, the first subgroup of each treatment was sampled to measure biomass and ZR content. The remaining subgroups in the BN group received only water under limited rewatering conditions, while those in the BN-1 to BN-5 groups were rewatered with nitrate (NO₃⁻) solutions at concentrations of 10, 20, 30, 40, and 50 mmol·L⁻¹, respectively. Biomass and ZR content were subsequently measured at 5 and 10 days after rewatering using the second and third subgroups, respectively.

#### Experiment 2

In Experiment 2, sixty pots were divided into five groups, each containing twelve pots. each receiving a distinct treatment: (1) continuous water supply (TA); (2) sufficient rewatering after drought (TB); (3) limited rewatering after drought (DB); (4) limited rewatering after drought with 27 mmol·L^− 1^ NO_3_^−^ solution (DN); and (5) limited rewatering after drought with 100 mL of *S2_8_1* bacterial solution (DJ). The TA treatment maintained a continuous and sufficient water supply throughout the experiment. In contrast, the TB treatment underwent a drought phase followed by sufficient rewatering, representing drought stress and rewatering phases, respectively. Similarly, the DB, DN, and DJ treatments experienced drought but were subjected to limited rewatering, also corresponding to the drought stress and limited rewatering phases.

Each treatment group (12 pots) was divided into four subgroups of three pots each, with each pot serving as a biological replicate. Biomass was measured at the end of the wet period using the first subgroup. At the onset of rewatering, all indicators—including biomass, ZR content, and others shown in Fig. 1—were assessed using the second subgroup. During rewatering, the third and fourth subgroups in the DJ treatment received a liquid medium containing the HAOB strain, followed by water to maintain soil moisture. In contrast, subgroups in the DB, TB, and TA treatments received the same medium without the HAOB strain, also followed by water. For the DN treatment, the third and fourth subgroups were continuously supplied with NO₃⁻ solution throughout the rewatering phase. All indicators were measured again at 5 and 10 days after rewatering using the third and fourth subgroups, respectively.

#### Soil moisture regulation

Previous research [21] has identified soil moisture levels of 75 ± 5%, 60 ± 5%, and 45 ± 5% of field capacity as thresholds for wet, moderate drought, and severe drought conditions, respectively. Building on these findings, our study defined three moisture treatments: sufficient water supply (75 ± 5%), limited water supply (60 ± 5%), and drought stress (45 ± 5%). A 10-day drought period was selected, as preliminary studies demonstrated its effectiveness in slowing maize seedling growth without inducing severe damage. To maintain target moisture levels, water was added whenever soil moisture dropped below the designated thresholds. Soil water content was calculated using Formula (1):

1 $$\:\text{S}\text{W}\text{C}=\frac{{\text{B}}_{\text{t}}-{\text{B}}_{\text{d}}-{\text{B}}_{\text{e}}-{\text{B}}_{\text{p}}}{{\text{B}}_{\text{d}}\times\:\text{F}\text{W}\text{C}}\times\:100{\%}$$

Here, *SWC* refers to soil moisture content, while *B*_*t*_, *B*_*d*_, *B*_*e*_, and *B*_*p*_ represent the total pot weight at a given time, dry soil mass, empty pot weight, and estimated live plant mass, respectively. *FWC* denotes the soil’s water-holding capacity. *B*_*p*_ was measured using extra pots during the experiment’s initial phase.

### Measurements

#### Biomass, water use efficiency

The soil attached to the roots was rinsed off with water, and the collected roots, stems, and leaves were oven-dried at 65 °C for 72 h to determine dry biomass. Aboveground biomass was calculated as the sum of stem and leaf dry masses, while total biomass included all three components. Water use efficiency (WUE) was calculated as the ratio of total biomass gain per pot to water consumption during the drought and rewatering phases. Biomass gain was determined by the difference in total biomass between the end of the well-watered phase and the end of the rewatering period, and water consumption was calculated by summing all water added throughout the drought stress and rewatering periods. Net photosynthetic rate (*P*_*n*_), stomatal conductance (*S*_*c*_), and transpiration rate (*T*_*r*_) were measured from 10:00 am to 1:00 pm using a *LI-6800* Photosynthesis Analyzer, under a photosynthetically active photon flux density ranging from 1300 to 1900 µmol·m⁻²·s⁻¹.

#### Soil nitrification rate

Cut open the pots and extract the roots along with the surrounding soil. Shake the roots repeatedly until only fine soil grains remain, which are collected as rhizosphere soil samples for analysis. Soil NO_3_^−^ and NH_4_^+^ contents were determined using the phenol disulfonic acid and indophenol blue colorimetric methods, respectively [22]. To assess daily NO_3_^−^ accumulation, soil samples adjusted to 60% of field capacity were incubated at 25 °C for a week, from which the rates of nitrification were determined.

#### Zeatin riboside

The ZR, a stable form of zeatin, is essential for cytokinin communication and metabolic processes. It was consistently detected in plant vascular tissues, including leaves, stems, and roots. A strong positive correlation was found between ZR content in maize leaves and rapid growth after rewatering following drought stress, making it a key indicator of cytokinin levels [23]. An enzyme-linked immunosorbent assay was used to measure the ZR concentrations in leaves and xylem sap.

Maize stems were cut from the roots, and a 1.0 g cotton wadding was placed over the incision to absorb xylem sap for 12 h. To minimize evaporation, the cotton was wrapped in plastic film. The absorbed sap’s volume was estimated by dividing the cotton’s weight increase by the density of water (1 g·cm⁻³). This volume per unit time reflected the transport rate from roots to leaves. The collected cotton was then squeezed multiple times to extract the sap, which was analyzed for ZR concentration (*C*_*ZR*_). Both *C*_*ZR*_ and leaf ZR content were quantified using commercial ZR assay kits (Shanghai Enzyme-linked Biotechnology Co., Ltd., Shanghai, China).

The ZR content in leaves was quantified as the amount per unit leaf mass, while *C*_*ZR*_ was determined based on ZR per unit sap volume. Root-to-leaf ZR transport was evaluated under two conditions: in darkness (*B*_*ZR*_) and under light (*L*_*ZR*_), using the following equations as described by Wang et al., [24].

2 $$\:\text{B}\text{Z}\text{R}={C}_{zr}\times\:{X}_{r}$$

3 $$\:{\text{L}}_{\text{Z}\text{R}}={C}_{zr}\times\:{T}_{r}\times\:M\times\:SLA$$

Here, *X*_*r*_ represents the xylem sap transport rate in darkness, *T*_*r*_ indicates the plant transpiration rate, *M* refers to leaf biomass, and *SLA* (Specific Leaf Area) denotes the ratio of leaf surface area to dry mass.

#### Data analysis

Statistical analysis was conducted using SPSS 27. The values shown in the graphs denote the means. One-way ANOVA followed by Duncan’s multiple range test (*P* = 0.05) was used to evaluate soil nitrification rate, biomass, hormone levels, nitrogen indices, and other relevant parameters. Bar charts were created with Excel (Microsoft 2024).

## Results

### Experiment 1

**Fig. 2:**
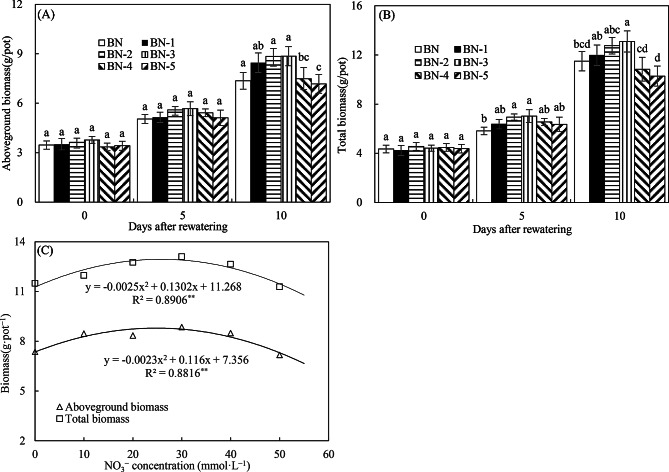
Aboveground and total biomasses (**A**, **B**) and their correlation with NO₃⁻ concentrations (**C**) in Experiment 1. The time points “0”, “5”, and “10” correspond to the start, middle, and end of the 10-day rewatering period. Treatments BN to BN-5 represent rewatering alone or combined with 10–50 mmol·L⁻¹ NO₃⁻ supplementation. Data are presented as mean ± standard error (*n* = 3), with different lowercase letters denoting significant differences (*P* < 0.05)

Figure 2A and B demonstrate a clear upward trend in aboveground and total biomass from BN-1 to BN-3, with each treatment producing greater biomass than the BN control at both 5 and 10 days after limited rewatering. Notably, BN-3 (30 mmol·L⁻¹ NO₃⁻) resulted in significantly higher biomass under these conditions. Regression analysis (Fig. 2C) further revealed that the optimal NO₃⁻ concentration for promoting biomass accumulation under limited rewatering after drought lies between 20 and 30 mmol·L⁻¹, with a predicted maximum at 26.04 mmol·L⁻¹.

**Fig. 3:**
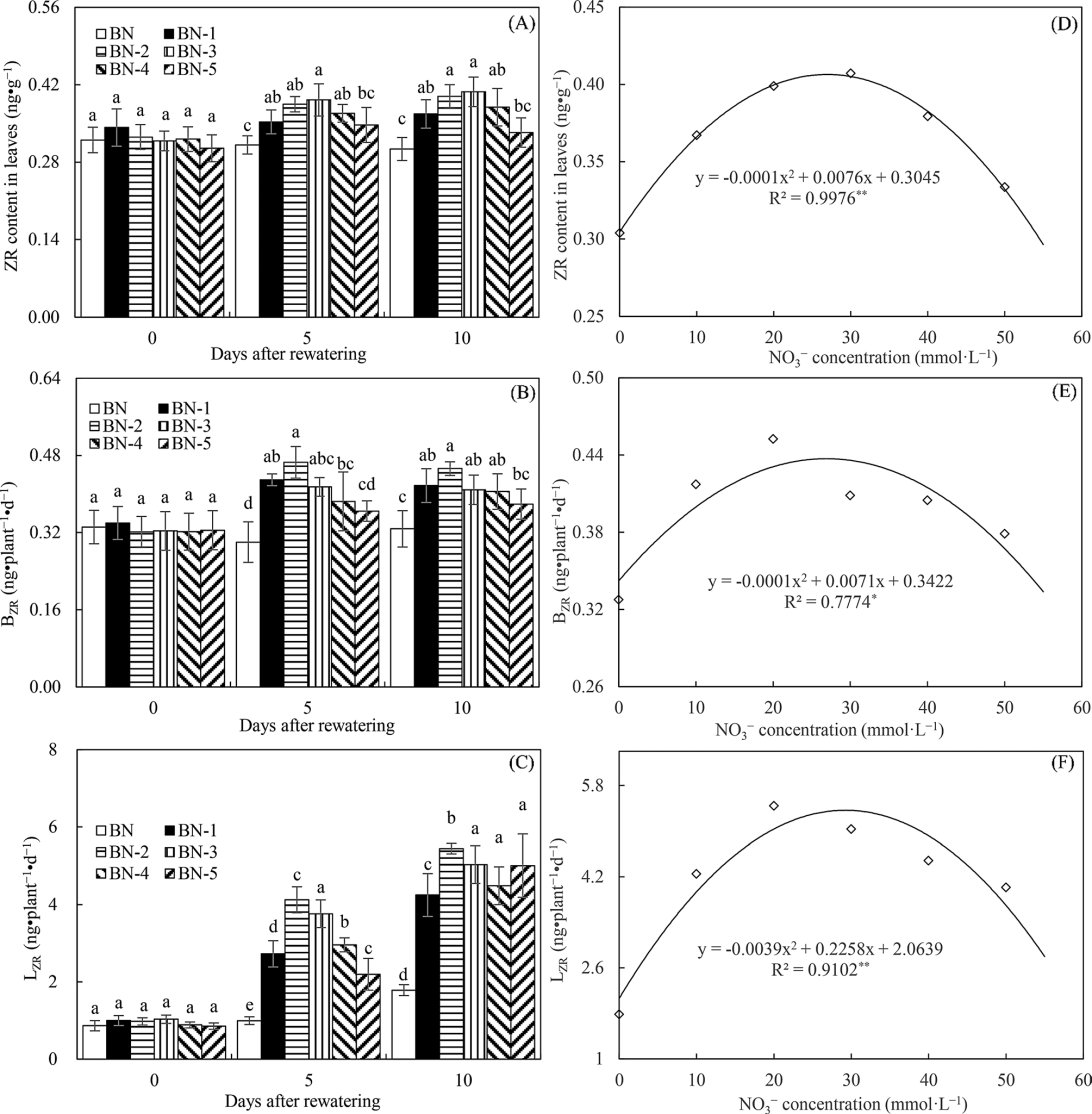
ZR content in leaves (**A**), B_ZR_ (**B**), L_ZR_ (**C**), and their relationship with NO_3_^−^ concentrations (**D**, **E**, **F**) in Experiment 1. The time points “0”, “5”, and “10” correspond to the start, middle, and end of the 10-day rewatering period. Treatments BN to BN-5 represent rewatering alone or combined with 10–50 mmol·L⁻¹ NO₃⁻ supplementation. ZR represents the cytokinin zeatin riboside; B_ZR_ and L_ZR_ represent the root-to-leaf ZR transport rate during dark and light periods, respectively. Data are presented as mean ± standard error (*n* = 3), with different lowercase letters denoting significant differences (*P* < 0.05)

Figure 3A shows that treatments BN-1 to BN-4 significantly increased leaf ZR content compared to BN at both 5 and 10 days after limited rewatering. Similar trends were observed for *B*_*ZR*_ and *L*_*ZR*_ (Fig. 3B and C). As a key cytokinin, elevated ZR levels suggest that NO₃⁻ enhances cytokinin accumulation in leaves and promotes its translocation from roots to shoots. Regression analysis further indicated that NO₃⁻ concentrations of 27.14, 26.69, and 28.89 mmol·L⁻¹—all within the 20–30 mmol·L⁻¹ range—were optimal for increasing leaf ZR, *B*_*ZR*_, and *L*_*ZR*_ levels under limited rewatering conditions (Fig. 3D–F).

### Experiment 2

**Fig. 4:**
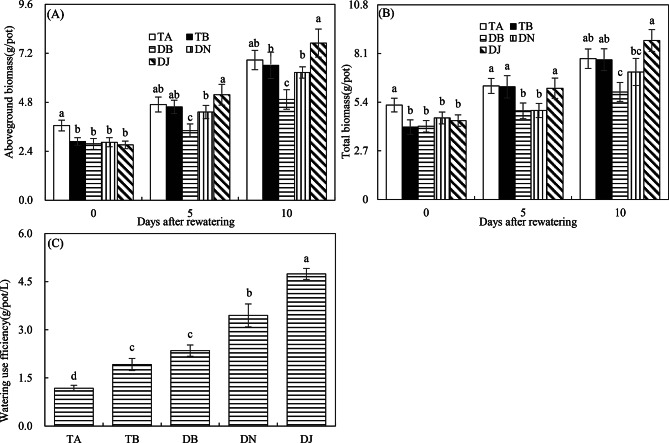
Aboveground and total biomasses (**A**, **B**) and Water use efficiency (**C**) in Experiment 2. The time points “0”, “5”, and “10” correspond to the start, middle, and end of the 10-day rewatering period. Treatments TA and TB correspond to regular water supply, post-drought sufficient rewatering, respectively. DB, DN, and DJ denote post-drought limited rewatering alone, with 27 mmol·L⁻¹ NO₃⁻, or with 100 mL of S2_8_1 bacterial solution, respectively. Data are presented as mean ± standard error (*n* = 3), with different lowercase letters denoting significant differences (*P* < 0.05)

#### Biomass

Figure 4A and B show that at the end of the drought period, the TA treatment resulted in the highest aboveground and total biomass among all treatments, respectively, highlighting the substantial impact of drought stress on maize growth. Ten days after rewatering, TA and TB exhibited comparable aboveground and total biomasses, whereas these values remained significantly lower in the DB treatment compared to TA. These results suggest that sufficient rewatering facilitates compensatory growth, while limited rewatering constrains it.

Compensatory growth occurred under limited rewatering when combined with NO₃⁻ addition and HAOB inoculation, as evidenced by the comparable total biomass in the DN and TA treatments, as well as in the DJ and TA treatments on the 10th day after rewatering (Fig. 4B). Notably, by the end of the rewatering period, total biomass in DJ was 25% higher than in DN, highlighting that HAOB inoculation is more effective than NO₃⁻ addition alone in promoting maize growth under limited rewatering conditions.

The DJ treatment achieved the highest water use efficiency (WUE), followed by DN. DB and TB ranked next, while TA had the lowest WUE (Fig. 4C). These results highlight the significant improvement in WUE driven by both NO₃⁻ and HAOB strains. Notably, HAOB strains proved more effective than NO₃⁻ in enhancing WUE under limited rewatering conditions.

#### Photosynthesis

**Fig. 5:**
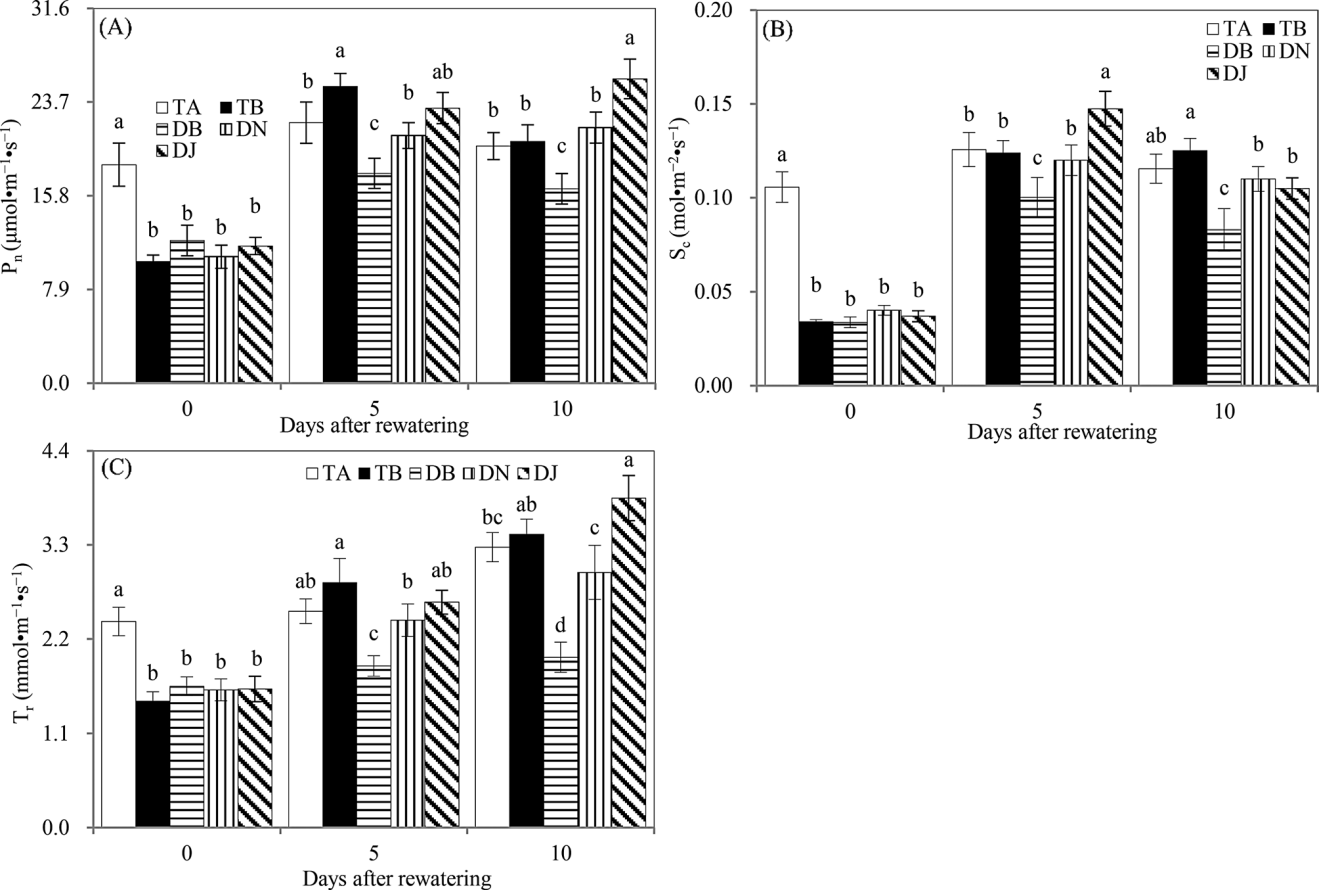
Photosynthesis rate (P_n_, (**A**)), stomatal conductance (S_c_, (**B**)), and transpiration rate (T_r_, (**C**)) in Experiment 2. The time points “0”, “5”, and “10” correspond to the start, middle, and end of the 10-day rewatering period. Treatments TA and TB correspond to regular water supply, post-drought sufficient rewatering, respectively. DB, DN, and DJ denote post-drought limited rewatering alone, with 27 mmol·L⁻¹ NO₃⁻, or with 100 mL of S2_8_1 bacterial solution, respectively. Data are presented as mean ± standard error (*n* = 3), with different lowercase letters denoting significant differences (*P* < 0.05)

Prior to rewatering, the TA treatment showed significantly higher *P*_*n*_, *T*_*r*_, and *S*_*c*_ values than other treatments, indicating that drought stress suppressed maize photosynthesis (Fig. 5A–C). Sufficient rewatering further enhanced photosynthesis, while limited rewatering hindered its recovery. This is evident from the significant increases in *P*_*n*_, *T*_*r*_, and *S*_*c*_ in TB compared to TA, and in TA compared to DB, on days 5 after rewatering. On day 10 post-rewatering, the DJ treatment exhibited higher *P*_*n*_, *T*_*r*_, and *S*_*c*_ values than TA, whereas DN showed similar levels to TA. These findings suggest that the HAOB strain improved maize photosynthesis under limited rewatering and was more effective than NO₃⁻ in promoting photosynthesis under such conditions.

#### Rhizosphere soil nitrification

**Fig. 6:**
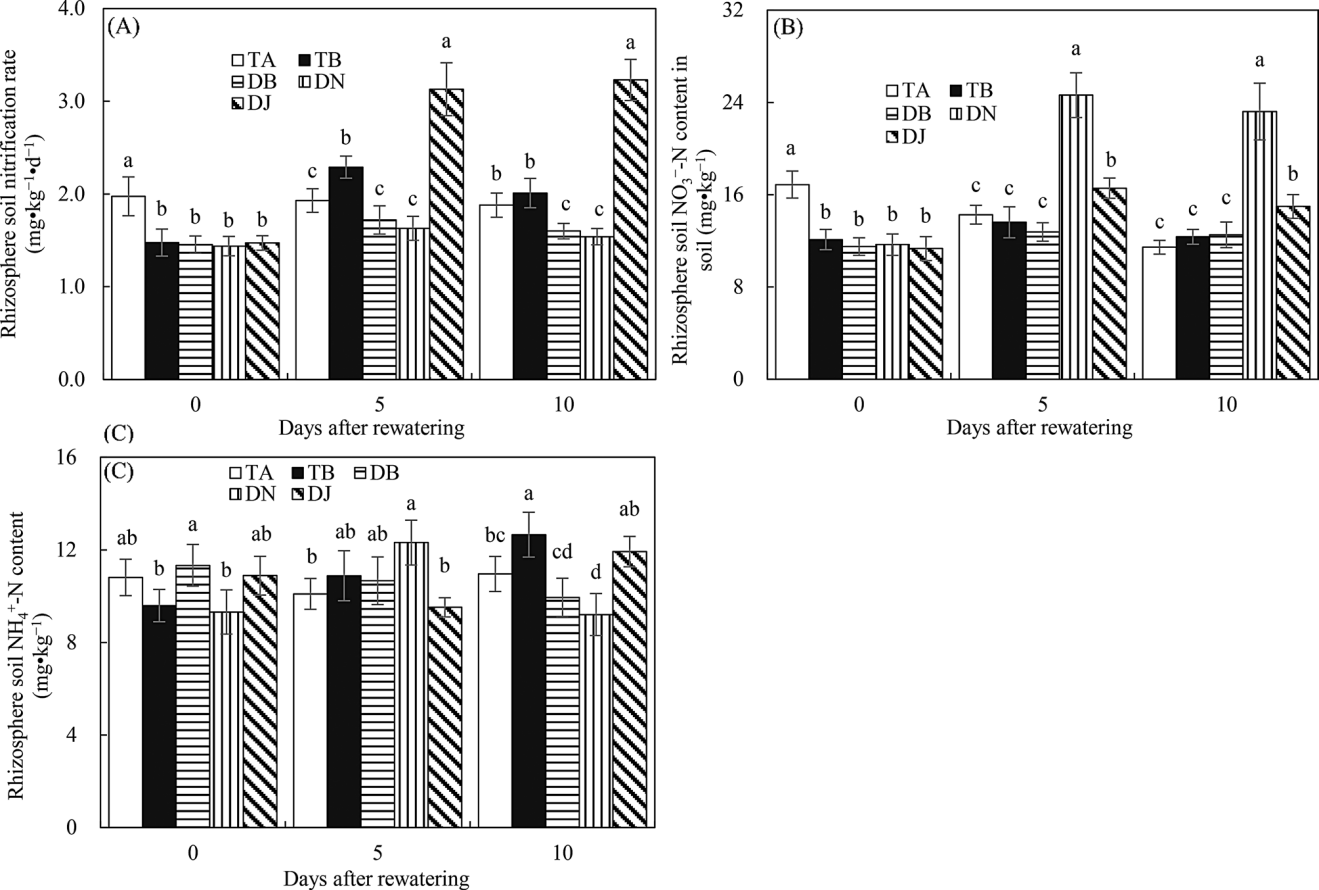
Rhizosphere soil nitrification rates (**A**), NO_3_^−^-N contents (**B**), and NH_4_^+^-N contents (**C**) in Experiment 2. The time points “0”, “5”, and “10” correspond to the start, middle, and end of the 10-day rewatering period. Treatments TA and TB correspond to regular water supply, post-drought sufficient rewatering, respectively. DB, DN, and DJ denote post-drought limited rewatering alone, with 27 mmol·L⁻¹ NO₃⁻, or with 100 mL of S2_8_1 bacterial solution, respectively. Data are presented as mean ± standard error (*n* = 3), with different lowercase letters denoting significant differences (*P* < 0.05)

Figure 6A shows that drought stress suppressed soil nitrification, as evidenced by the significantly higher nitrification rate in the TA treatment compared to others before rewatering. This inhibition was alleviated by sufficient rewatering, with TB exhibiting a significantly higher nitrification rate than TA. Notably, on days 5 and 10 after rewatering, the DJ treatment maintained nitrification rates at least 1.4 times higher than other treatments, indicating that the HAOB strain effectively promoted nitrification even under limited rewatering conditions.

As shown in Fig. 6B, during the rewatering phase, rhizosphere soil NO₃⁻ content in the DN treatment was at least 1.5 times higher than in other treatments, while DJ maintained levels at least 1.2 times higher than TA, TB, and DB, suggesting that both NO₃⁻ addition and HAOB inoculation positively influenced NO₃⁻ availability. In contrast, Fig. 6C indicates that NH₄⁺ concentrations varied inconsistently across treatments during rewatering, implying that neither HAOB inoculation, rewatering, nor NO₃⁻ addition significantly increased NH₄⁺ levels.

#### Zeatin riboside

**Fig. 7:**
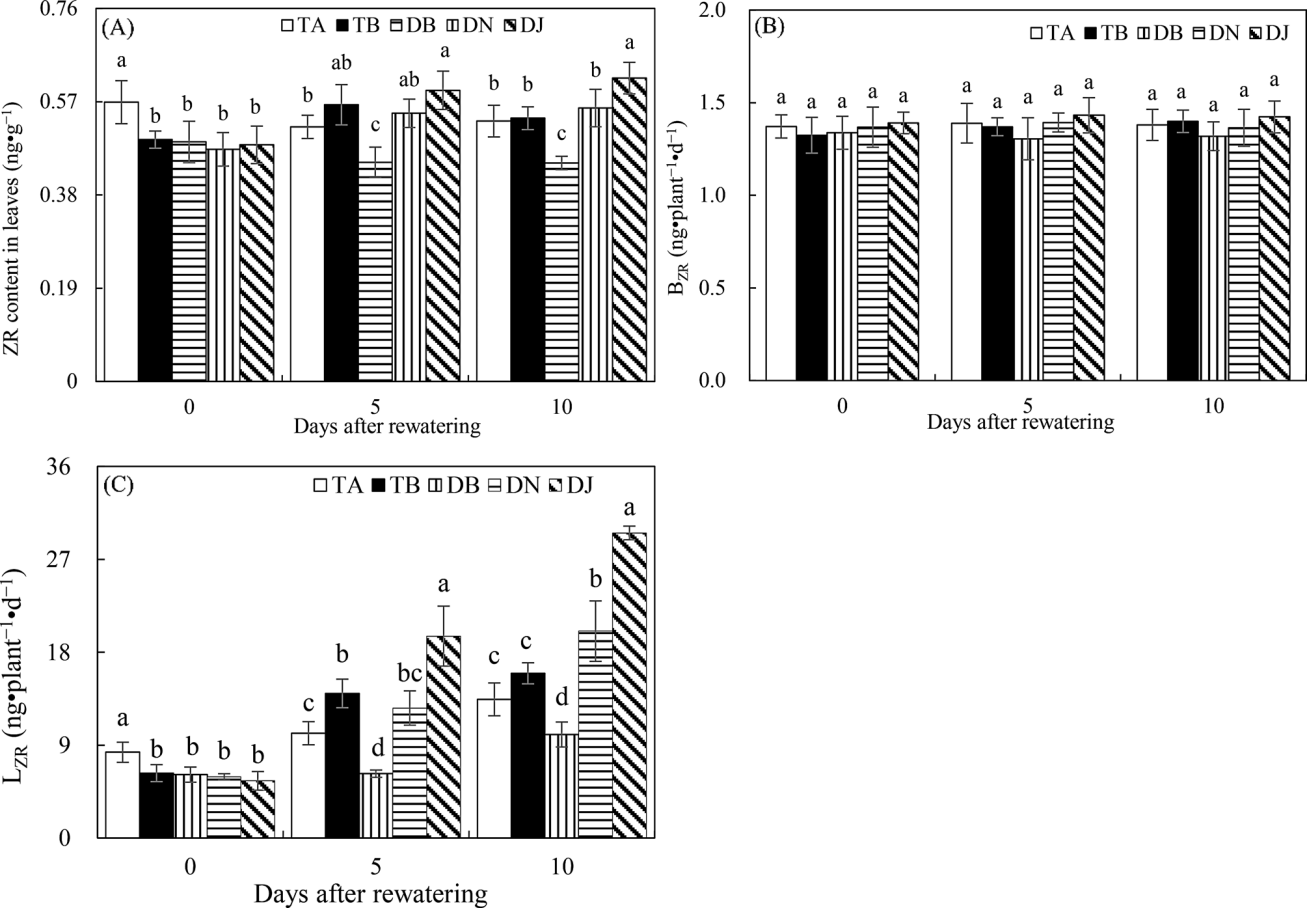
ZR content in leaves (**A**), B_ZR_ (**B**), and L_ZR_ (**C**) in Experiment 2. The time points “0”, “5”, and “10” correspond to the start, middle, and end of the 10-day rewatering period. Treatments TA and TB correspond to regular water supply, post-drought sufficient rewatering, respectively. DB, DN, and DJ denote post-drought limited rewatering alone, with 27 mmol·L⁻¹ NO₃⁻, or with 100 mL of S2_8_1 bacterial solution, respectively. Data are presented as mean ± standard error (*n* = 3), with different lowercase letters denoting significant differences (*P* < 0.05)

Drought conditions reduce cytokinin levels in leaf cells and impede their translocation from roots to shoots. As ZR, a major cytokinin type, by the end of the drought period, the TA treatment exhibited significantly higher leaf ZR content and root-to-shoot translocation rates (*L*_*ZR*_ and *B*_*ZR*_) than other treatments (Fig. 7A–C). Moreover, sufficient rewatering elevated cytokinin levels in leaves and enhanced its movement from roots to shoots compared to limited rewatering and sufficient water supply. This was reflected in the significantly higher ZR content in leaves and *L*_*ZR*_ in TB than in DB on days 5 and 10 after rewatering, with similar trends observed when comparing TB with TA during the same period.

At day 10 after rewatering, ZR content in leaves and *L*_*ZR*_ decreased significantly in DB compared to DN and DJ, and in DJ compared to other treatments (Fig. 7A and C). These findings suggest that NO₃⁻ supplementation and HAOB strain inoculation enhanced cytokinin levels in leaves and promoted root-to-leaf translocation under limited rewatering. Notably, the HAOB strain had a stronger effect than NO₃⁻, as evidenced by significantly higher leaf ZR content in DJ compared to DN on day 10 post-rewatering, with a similar trend observed for *B*_*ZR*_ and *L*_*ZR*_ (Fig. 7A–C).

## Discussion

### HAOB strain-mediated dual roles

Under limited rewatering, inoculating the HAOB strain in the DJ treatment increased the rhizosphere soil nitrification rate. This rate was at least 1.5 times higher than that of the DB, TB, and TA treatments. As a result, rhizosphere NO_3_^−^ content also increased by at least 1.2-fold. Elevated NO₃⁻ availability enhanced root cytokinin production. It also enhanced their translocation to the leaves, as evidenced by the increased *B*_*ZR*_ and *L*_*ZR*_ levels. This resulted in about a 15% higher leaf cytokinin content in the DJ treatment than in TA during rewatering. The apical meristem at the root tip, essential for cytokinin production and nutrient uptake, showed increased cytokinin synthesis when stimulated by NO_3_^−^ [21, 23–25]. Cytokinins are recognized for their roles in enhancing photosynthesis, cell division, elongation, and the accumulation of organic matter [26–28], which likely accounts for the elevated cytokinin levels in leaves in DJ and the subsequent robust plant growth during rewatering. As a result, the *P*_*n*_ in the DJ treatment increased by 17% compared to TA during rewatering. By the end of the rewatering period, total biomass in DJ was 13% higher than in TA, demonstrating compensatory growth.

In the DJ treatment, *L*_*ZR*_ increased by 50%, *B*_*ZR*_ by 4%, and leaf cytokinin content by 9% compared to DN during rewatering, which lead to 25% higher total biomass by the end of the rewatering period. These increases cannot be solely attributed to NO_3_^−^ from HAOB strain-facilitated nitrification, given the chosen NO₃⁻ concentration (27 mmol·L^− 1^) in the DN treatment was sufficient to promote root cytokinin synthesis. In the Experiment 2, the most effective promotion of ZR content in leaves, *B*_*ZR*_, *L*_*ZR*_, and total biomass by NO_3_^−^ occurred within the 20–30 mmol·L^− 1^ range, with optimal concentrations at 27.14, 26.69, 28.95, and 26.04 mmol·L^− 1^, respectively. Therefore, a NO_3_^−^ concentration of 27 mmol·L^− 1^ closely matches the optimal enhancement of root cytokinin moving to foliage and its subsequent impact on growth during rewatering. It is well-established that rhizosphere bacteria can actively regulate cytokinin synthesis in host plants [29, 30]. Thus, in the DJ treatment, the HAOB strain likely enhances root cytokinin production independently of nitrification, highlighting its twofold-function mechanism.

### Twofold-function in promoting compensatory growth and water use

Sufficient rewatering after drought increased nitrification in the maize rhizosphere, with the TB treatment showing a 13% higher nitrification rate than TA during rewatering. This increase facilitated root-derived cytokinin translocation to the shoot, promoting compensatory growth in TB, consistent with findings by Wang et al. [31]. Conversely, limited rewatering inhibited rhizosphere soil nitrification, evidenced by a 15% lower soil nitrification rate in DB compared to TA at the conclusion of the rewatering phase, disrupting cytokinin transport and preventing compensatory growth. In the DN treatment, despite over 80% higher NO_3_^−^ content in the rhizosphere soil compared to TB and TA during rewatering, limited rewatering conditions hindered root cytokinin synthesis and transport, resulting in about 10% less total biomass compared to TA or TB by the end of rewatering period. This outcome highlights the critical role of water availability in cytokinin synthesis and transport, as also noted by Paul et al. [32] and Todaka et al. [33].

The DJ treatment, utilizing dual mechanisms, successfully overcame water limitations, leading to a 13% increase in biomass compared to the TA treatment by the end of the rewatering phase. Specifically, while a limited water supply can hinder cytokinin transport through NO_3_^−^ release from HAOB strain-mediated soil nitrification, the HAOB strain compensates by directly promoting cytokinin transport from the roots. Forni et al. [34] and Mekureyaw et al. [35] have highlighted the importance of cytokinins from rhizosphere bacteria in enhancing plant growth under drought stress. Consequently, this dual mechanism significantly improves water use efficiency (WUE) in maize, with the DJ treatment achieving 50% higher WUE than DN, 150% higher than in TB, and 180% higher than in TA.

### Significance of the dual-role mechanism

The application of nitrogen fertilizer is a well-established agronomic practice for enhancing drought resistance and water use efficiency in crops [36]. However, our study reveals certain limitations of nitrogen fertilizer in effectively enhancing drought resistance and water use efficiency in maize. Under limited rewatering conditions, the dual action of nitrogen enhancement and cytokinin production mediated by the HAOB strain effectively overcomes these limitations, leading to improved compensatory growth and water use efficiency. This suggests that future development of new water-saving agricultural technologies based on this dual-action mechanism holds significant potential and could greatly contribute to enhancing agricultural productivity.

The primary method for using cytokinins in field crops typically involves foliar spraying of exogenously synthesized cytokinins, and this approach is mostly confined to small-scale experimental trials [37, 38]. The large-scale application of cytokinins to enhance drought resistance and promote water-saving growth in crops remains uncommon. This study is the first to achieve cytokinin regulation through microbial inoculation—biotechnology, presenting a novel approach for developing new agricultural technologies aimed at the widespread application of cytokinins for improving crop resilience to drought in large-scale agriculture.

Previous studies have shown that metabolic products of many soil bacteria can influence hormone levels in plant roots, particularly cytokinins—a trait widely observed among soil microorganisms [39]. In our preliminary experiments, the HAOB strain *S2_8_1* was found to produce measurable amounts of cytokinins in its metabolites (see Materials and Methods for details). Rhizosphere bacteria capable of producing cytokinins can regulate both their concentration and biosynthesis in host plants [29, 30]. Therefore, the non-nitrification pathway in the dual-function mechanism proposed in this study is likely linked to the HAOB strain’s inherent ability to synthesize cytokinins. Further research should explore this pathway in greater depth to fully uncover the mechanisms by which HAOB strains enhance crop growth.

## Conclusion

This study demonstrates that NO_3_^−^ addition significantly boosts maize growth and cytokinin levels in leaves, with optimal concentrations of 20–30 mmol·L^− 1^ under limited rewatering. The HAOB strain plays a crucial role in enhancing rhizosphere soil nitrification and promoting cytokinin translocation from roots to shoots, especially under limited rewatering conditions. Cytokinin translocation from roots to leaves, facilitated by HAOB strains, operates through two pathways: nitrification and a direct route. While sufficient rewatering without HAOB inoculation enhances growth by stimulating nitrification, which promotes cytokinin translocation and enables compensatory growth, limited rewatering without HAOB inoculation restricts these processes, hindering compensatory growth. HAOB strains overcome this limitation by directly enhancing cytokinin translocation, compensating for the nitrification pathway’s deficiencies, achieving compensatory growth, and improving water use efficiency. This dual mechanism significantly elevates cytokinin levels in leaves and supports compensatory growth even under limited water conditions.

## Data Availability

All data are available from the corresponding author upon reasonable request.

## Abbreviations

BN: Limited rewatering alone for determining the optimal NO_3_^−^ concentration
BN-1: Limited rewatering with 10 mmol·L^− 1^ NO_3_^−^
BN-2: Limited rewatering with 20 mmol·L^− 1^ NO_3_^−^
BN-3: Limited rewatering with 30 mmol·L^− 1^ NO_3_^−^
BN-4: Limited rewatering with 40 mmol·L^− 1^ NO_3_^−^
BN-5: Limited rewatering with 50 mmol·L^− 1^ NO_3_^−^
TA: Continuous sufficient water supply
TB: Sufficient rewatering after drought alone
DB: Limited rewatering after drought alone
DN: Limited rewatering after drought with 27 mmol·L^− 1^ NO_3_^−^ solution
DJ: Limited rewatering after drought with 100 mL of S2_8_1 bacterial solution
NO_3_^−^: Nitrate
NH_4_^+^: Ammonium
HAOB: Heterotrophic ammonia-oxidizing bacteria
DMPP: 3,4-dimethylpyrazole phosphate
SWC: Soil water content
B_t_: Temporary weight of the whole pot
B_d_: Net weight of dried soil of pot
B_e_: Weight of the empty pot
B_p_: Estimated live plant mass
FWC: Field water capacity
WUE: Water use efficiency
P_n_: Photosynthetic rate
S_c_: Stomatal conductance
T_r_: Transpiration rate
ZR: Zeatin riboside
R_ZR_: Delivery rate of ZR from roots to leaves
C_ZR_: ZR concentration in xylem sap
IAA: Indole-3-acetic acid
GA_3_: Gibberellic acid
X_r_: Xylem sap transport rate in darkness
M: Leaf biomass
SLA: Specific Leaf Area

## References

[1] Belsky AJ. Does herbivory benefit plants—a review of the evidence. Am Nat. 1986;127(6):870–92.

[2] Wang XL, Wang JJ, Sun RH, Hou XG, Zhao W, Shi J, Zhang YF, Qi L, Li XL, Dong PH, Zhang LX, Xu GW, Gan HB. Correlation of the corn compensatory growth mechanism after post-drought rewatering with cytokinin induced by root nitrate absorption. Agr Water Manage. 2016;166:77–85.

[3] Zhang JX, Wang QQ, Xia GM, Wu Q, Chi DC. Continuous regulated deficit irrigation enhances peanut water use efficiency and drought resistance. Agr Water Manage. 2021;255:106997.

[4] Himanshu SK, Ale S, Bordovsky J, Darapuneni M. Evaluation of crop-growth-stage-based deficit irrigation strategies for cotton production in the Southern high plains. Agr Water Manage. 2019;225:105708.

[5] Zhang HB, Han K, Gu SB, Wang D. Effects of supplemental irrigation on the accumulation, distribution and transportation of 13 C-photosynthate, yield and water use efficiency of winter wheat. Agr Water Manage. 2019;214:1–8.

[6] Kang SZ, Hao XM, Du TS, Tong L, Su XL, Lu HN, Li XL, Huo ZL, Li SE, Ding RS. Improving agricultural water productivity to ensure food security in China under changing environment: from research to practice. Agr Water Manage. 2017;179:5–17.

[7] Li HF, Qi ZM, Gui DW, Zeng FJ. Water use efficiency and yield responses of cotton to field capacity-based deficit irrigation in an extremely arid area of China. Int J Agric Biol Eng. 2019;12(6):91–101.

[8] Wang XL, Qin RR, Sun RH, Wang JJ, Hou XG, Qi L, Shi J, Li XL, Zhang YF, Dong PH, Zhang LX, Qin DH. No post-drought compensatory growth of corns with root cutting based on cytokinin induced by roots. Agr Water Manage. 2018;205:9–20.

[9] Wang XL, Duan PL, Sun RH, Qi L, Shi J, Li XL, Zhang LX. Effects of soil nitrification on compensatory growth upon post-drought rewatering of corns based on cytokinin. Int J Agric Biol. 2020;23(5):882–8.

[10] Qin RR, Wang XL. Effects of crown height on the compensatory growth of Italian ryegrass based on combined effects of stored organic matter and cytokinin. Grassl Sci. 2020;66:29–39.

[11] Barragán-Fonseca KY, Nurfikari A, van de Zande EM, Wantulla M, van Loon JJA, Boer WD, Dicke M. Insect Frass and exuviae to promote plant growth and health. Trends Plant Sci. 2022;27:646–54. 35248491 10.1016/j.tplants.2022.01.007

[12] Li HR, Li XL, Mei XR, Nangia V, Guo R, Hao WP, Wang JD. An alternative water-fertilizer-saving management practice for wheat-maize cropping system in the North China plain: based on a 4-year field study. Agr Water Manage. 2023;276:108053.

[13] Wang XL, Sun RH, Wu D, Qi L, Shi J, Li XL, Song P, Zhang LX. Increasing corn compensatory growth upon post-drought rewatering using ammonia-oxidising bacterial strain inoculation. Agr Water Manage. 2021;256:107066.

[14] Wang XL, Ma K, Qi L, Liu YH, Shi J, Li XL, Zhang LX, Liu W, Song P. Effect of ammonia-oxidizing bacterial strain that survives drought stress on corn compensatory growth upon post-drought rewatering. Front Plant Sci. 2022;13:947476. 36186022 10.3389/fpls.2022.947476 PMC9520602

[15] Khan W, Zhu Y, Khan A, Zhao L, Yang YM, Wang N, Hao M, Ma Y, Nepal J, Ullah F, Rehman MM, U, Abrar M, Xiong YC. Above-and below-ground feedback loop of maize is jointly enhanced by plant growth-promoting rhizobacteria and arbuscular mycorrhizal fungi in drier soil. Sci Total Environ. 2024;917:170417. 38280611 10.1016/j.scitotenv.2024.170417

[16] Abrar M, Zhu Y, Rehman MMU, Batool A, Duan HX, Ashraf U, Aqeel M, Gong XF, Peng YN, Khan W, Wang ZY, Xiong YC. Functionality of arbuscular mycorrhizal fungi varies across different growth stages of maize under drought conditions. Plant Physiol Biochem. 2024;213:108839. 38879986 10.1016/j.plaphy.2024.108839

[17] Adesemoye AO, Torbert HA, Kloepper JW. Enhanced plant nutrient use efficiency with PGPR and AMF in an integrated nutrient management system. Can J Microbiol. 2008;54(10):876–86. 18923557 10.1139/w08-081

[18] Gao ZZ, Wang C, Zhao JC, Wang KC, Shang MF, Qin YS, Bo XZ, Chen F, Chu QQ. Adopting different irrigation and nitrogen management based on precipitation year types balances winter wheat yields and greenhouse gas emissions. Field Crop Res. 2022;280:108484.

[19] Wu D, Wang XL, Zhu XH, Wang HH, Liu W, Qi L, Song P, Zhang MM, Zhao W. Effect of ammonia-oxidizing bacterial strains that coexist in rhizosphere soil on Italian ryegrass regrowth. Microorganisms. 2022;10(11):2122. 36363714 10.3390/microorganisms10112122 PMC9696852

[20] Wu D, Ma K, Wang XL, Qi L, Liu YH, Song P, Liu W, Zhang MM, Zhao W, Song CW. Increasing Italian ryegrass (*Lolium multiflorum* Lam.) regrowth via inoculation with an ammonia-oxidizing bacterial strain. Grassl Sci. 2023;69(1):51–64.

[21] Cortleven A, Leuendorf JE, Frank M, Pezzetta D, Bolt S, Schmülling T. Cytokinin action in response to abiotic and biotic stresses in plants. Plant Cell Environ. 2019;42:998–1018. 30488464 10.1111/pce.13494

[22] Lu RK. Analysis method of the soil agricultural chemistry. Beijing: China Agricultural Science and Technology; 1999.

[23] Wang XL, Liu D, Li ZQ. Effects of the coordination mechanism between roots and leaves induced by root-breaking and exogenous cytokinin spraying on the grazing tolerance of ryegrass. J Plant Res. 2012;125:407–16. 21748489 10.1007/s10265-011-0442-x

[24] Wang XL, Tian SS, Yu H, Sun RH, Qi L, Song P, Yang SJ. Enhanced Post-Drought compensatory growth and water utilization in maize via rhizosphere soil nitrification by heterotrophic Ammonia-Oxidizing Bacteria. Water. 2023;15:3933.

[25] Oldroyd GE, Leyser O. A plant’s diet, surviving in a variable nutrient environment. Science. 2020;368. 10.1126/science.aba0196 32241923

[26] Kobayashi H, Inoue S, Gyokusen K. Photosynthesis-nitrogen relationship in a Hinoki Cypress (*Chamaecyparis obtusa*) canopy: A comparison with Japanese Cedar (*Cryptomeria japonica*). Photosynthetica. 2012;50:317–20.

[27] De Moura FB, Da S, Vieira MR, Simões ADN, F da Silva SL, De Medeiros DC, Paes RDA, S, de Oliveira AA. C do nascimento AH, E. Júnior WS. Participation of cytokinin on gas exchange and antioxidant enzymes activities. Ind J Plant Physiol. 2017;22:16–29.

[28] Wu WQ, Du K, Kang XY, Wei HR. The diverse roles of cytokinins in regulating leaf development. Hortic Res-England. 2021;8:118. 10.1038/s41438-021-00558-3 PMC8167137 34059666

[29] Liu F, Xing S, Ma H, Du Z, Ma B. Cytokinin-producing, plant growth-promoting rhizobacteria that confer resistance to drought stress in *Platycladus orientalis* container seedlings. Appl Microbiol Biotechnol. 2013;97(20):9155–64. 23982328 10.1007/s00253-013-5193-2

[30] Kudoyarova G, Arkhipova T, Korshunova T, Bakaeva M, Loginov O, Dodd IC. Phytohormone mediation of interactions between plants and non-symbiotic growth-promoting bacteria under edaphic stresses. Front Plant Sci. 2019;10:1368. 31737004 10.3389/fpls.2019.01368 PMC6828943

[31] Wang XL, Si ZQ, Yu H, Qi L, Liu W, Shi J, Song P. Unveiling the dual role of heterotrophic ammonia-oxidizing bacteria: enhancing plant regrowth through modulating cytokinin delivery. Front Microbiol. 2023a;14:1268442. 37808285 10.3389/fmicb.2023.1268442 PMC10557131

[32] Paul S, Wildhagen H, Janz D, Polle A. Drought effects on the tissue- and cell-specific cytokinin activity in Poplar. Aob Plants. 2018;10(1):plx067. 29354257 10.1093/aobpla/plx067 PMC5767954

[33] Todaka D, Zhao Y, Yoshida T, Kudo M, Kidokoro S, Mizoi J, Kodaira KS, Takebayashi Y, Kojima M, Sakakibara H, Toyooka K, Sato M, Fernie AR, Shinozaki K, Yamaguchi-Shinozaki K. Temporal and Spatial changes in gene expression, metabolite accumulation and phytohormone content in rice seedlings grown under drought stress conditions. Plant J. 2017;90(1):61–78. 28019048 10.1111/tpj.13468

[34] Forni C, Duca D, Glick BR. Mechanisms of plant response to salt and drought stress and their alteration by rhizobacteria. Plant Soil. 2017;410:335–56.

[35] Mekureyaw MF, Pandey C, Hennessy RC, Nicolaisen MH, Liu FL, Nybroe O, Roitsch T. The cytokinin-producing plant beneficial bacterium *Pseudomonas fluorescens* G20-18 primes tomato (*Solanum lycopersicu*m) for enhanced drought stress responses. J Plant Physiol. 2022;270:153629. 35151004 10.1016/j.jplph.2022.153629

[36] Zhu J, Cai YM, Li X, Yang LY, Zhang YC. High-nitrogen fertilizer alleviated adverse effects of drought stress on the growth and photosynthetic characteristics of *Hosta ‘Guacamole*’. BMC Plant Biol. 2024;24:299. 38632552 10.1186/s12870-024-04929-5 PMC11025241

[37] Ren BZ, Zhu YL, Zhang JW, Dong ST, Liu P, Zhao B. Effects of spraying exogenous hormone 6-benzyladenine (6-BA) after waterlogging on grain yield and growth of summer maize. Field Crop Res. 2016;188:96–104.

[38] Huo ZG, Yang H. Application of exogenous 6-benzyladenine at the Silking-Stage improves the starch quality of waxy maize suffering from Post-Silking drought stress. Starch-Stärke. 2022;74:2100276.

[39] Stirk WA, van Staden J. Flow of cytokinins through the environment. Plant Growth Regul. 2010;62:101–16.

